# Dysregulation of deubiquitinases in gastric cancer progression

**DOI:** 10.3389/fonc.2024.1456710

**Published:** 2024-11-13

**Authors:** Zifan Xu, Zi Lei, Shilan Peng, Xiaonan Fu, Yuanyuan Xu, Guoqing Pan

**Affiliations:** First Affiliated Hospital of Kunming Medical University, Department of Pathology, Kunming, China

**Keywords:** gastric cancer, deubiquitinase, ubiquitination, DUB inhibitor, tumorigenesis

## Abstract

Gastric cancer (GC), characterized by a high incidence rate, poses significant clinical challenges owing to its poor prognosis despite advancements in diagnostic and therapeutic approaches. Therefore, a comprehensive understanding of the molecular mechanisms driving GC progression is crucial for identifying predictive markers and defining treatment targets. Deubiquitinating enzymes (DUBs), also called deubiquitinases, function as reverse transcriptases within the ubiquitin-proteasome system to counteract protein degradation. Recent findings suggest that DUB dysregulation could be a crucial factor in GC pathogenesis. In this review, we examined recent research findings on DUBs in the context of GC, elucidating their molecular characteristics, categorizations, and roles while also exploring the potential mechanisms underlying their dysregulation in GC. Furthermore, we assessed the therapeutic efficacy of DUB inhibitors in treating malignancies and evaluated the prevalence of aberrant DUB expression in GC.

## 1 Introduction

The ubiquitin-proteasome system (UPS) is a crucial post-translational modification mechanism in eukaryotic cells, comprising ubiquitin, E1 ubiquitin-activating enzyme, E2 ubiquitin-binding enzyme, E3 ubiquitin ligase, and the 26S proteasome (1, 2). Ubiquitination, a reversible post-translational modification, is integral to various biological processes, such as proteolysis, DNA damage response and repair, cell cycle regulation, and immune response (3). The UPS primarily operates through two distinct processes. First, during ubiquitination, ubiquitin molecules are attached to substrate proteins, serving as a molecular tag (4). This labeling facilitates subsequent recognition and targeting of the modified proteins for cleavage, degradation, and recycling by the 26S proteasome complex (4). In contrast, deubiquitination involves the enzymatic removal of ubiquitin molecules from ubiquitinated proteins, mediated by specific hydrolases known as deubiquitinases (DUBs) (5). Ubiquitination and deubiquitination exist in a constant state of dynamic equilibrium, wherein the interplay between ubiquitinases and DUBs determines the ubiquitination status of specific target proteins. This equilibrium renders protein ubiquitination a versatile and dynamic post-translational modification (6). Moreover, certain DUBs and their associated enzymes play a significant role in the modification and processing of various ubiquitin-like proteins, such as small ubiquitin-like modifier (SUMO) proteins (7). A prominent example of this is the sentrin/SUMO-specific protease, which is essential for the processing of SUMO precursors and conjugates (8).

**Figure 1:**
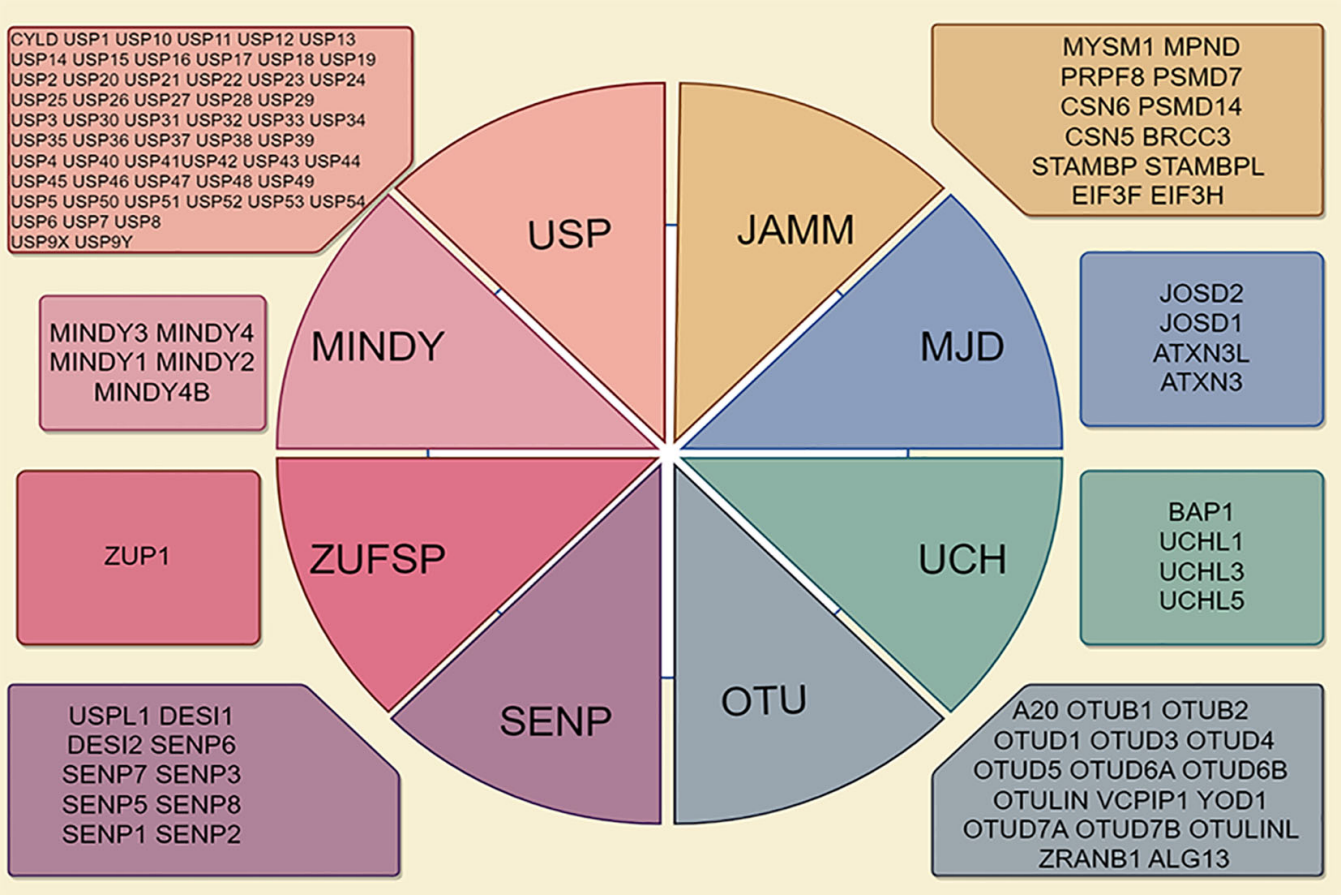
Deubiquitinase (DUB) family is characterized by its classification into seven distinct subfamilies, including ubiquitin-specific proteases (USPs), ubiquitin carboxy-terminal hydrolases (UCHs), ovarian tumor proteases (OTUs), Jab1/MPN domain-associated metalloenzymes (JAMMs), Machado-Joseph disease proteases (MJDs), monocyte chemotactic protein-induced protease family (MINDYs), and Zn-finger and UFSP domain proteins (ZUFSPs).

According to the principles of sequence and domain conservation, DUBs are categorized into six distinct families: ubiquitin-specific proteases (USPs), ubiquitin carboxyl-terminal hydrolases (UCHs), Machado-Josephin domain proteases, ovarian tumor proteases (OTUs), zinc finger proteases (ZUPs/ZUFSPs), and JAMM/MPN domain-associated metallopeptidases (JAMMs) (5, 9). Both sentrin/SUMO-specific proteases and the initial six DUB families are classified as cysteine peptidases, whereas JAMMs are categorized as zinc metallopeptidases (10, 11) (**Figure 1**). Among these families, the USP family is the largest and most diverse DUB group, characterized by a conserved catalytic domain comprising three subdomains resembling a right hand (12). Conversely, the UCH family, recognized as the first structurally characterized DUBs, possesses six or seven β-sheets and eight α-helices that obstruct larger substrates, thereby enabling the targeting of only small peptides from the C-terminus of ubiquitin (13). The OTU domain, initially identified in an ovarian tumor gene, includes five β-sheets situated between two helical domains (14). The Machado-Josephin domain protease family comprises four members: ATXN3, which is associated with Machado-Joseph disease, along with ATXN3L, JOSD1, and JOSD2 (15).

**Figure 2:**
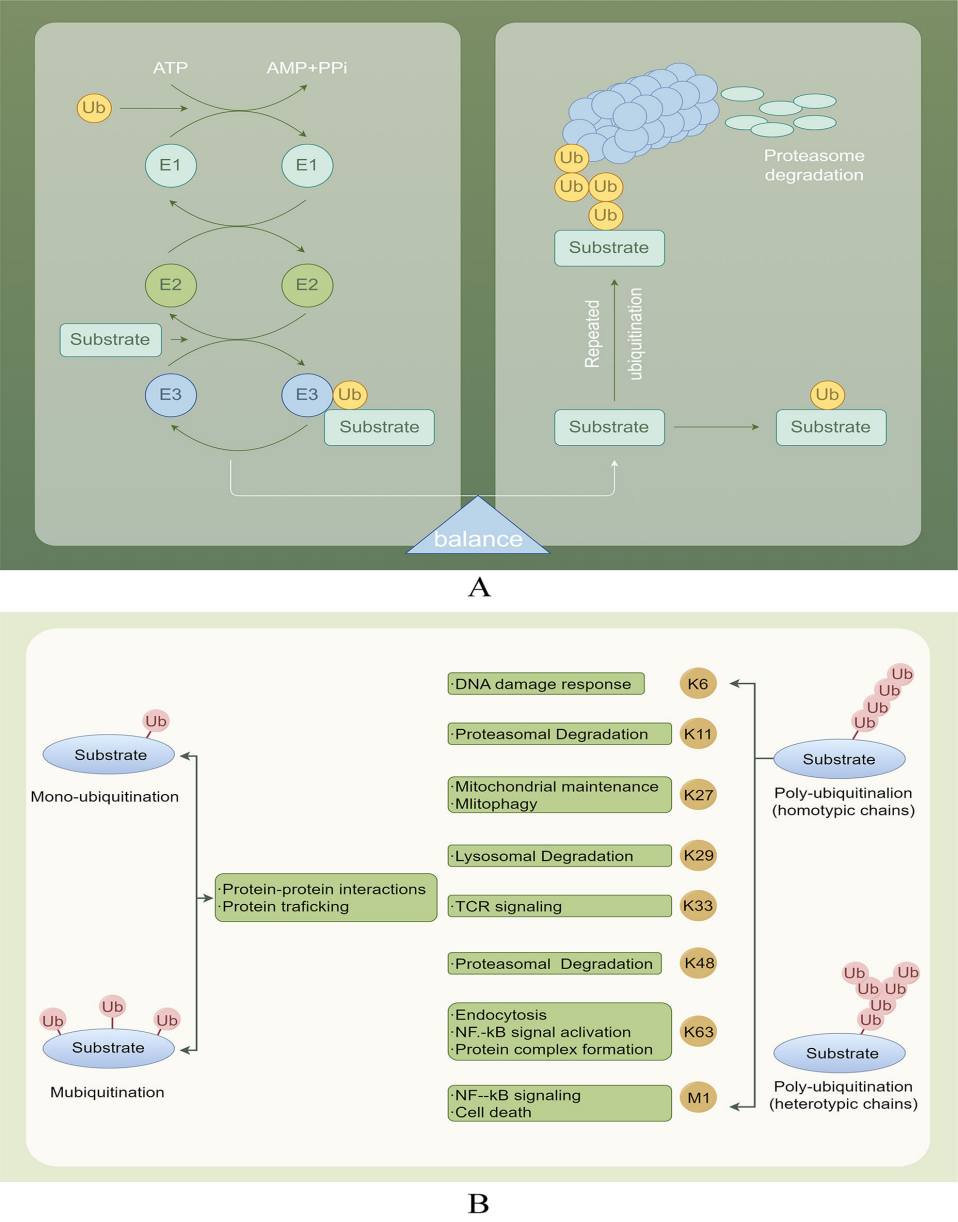
**(A)** Ubiquitination and deubiquitination involve attaching ubiquitin to E1, transferring it to E2, and using E3 ligase to deliver ubiquitin to target proteins. DUBs protect proteins by removing Ub and also help degrade labeled proteins through the proteasome, playing a role in physiological activities. **(B)** Various ubiquitination processes and their roles.

DUBs play a crucial role in the deubiquitination process, which involves the removal of ubiquitin chains from substrate proteins, thereby facilitating their recovery. Substrate proteins typically undergo multiple ubiquitination processes, resulting in the formation of single or multiple ubiquitin chains (**Figure 2A**). Ubiquitin can be conjugated to proteins at one or more lysines, resulting in monoubiquitination (a single molecule) or polyubiquitination (the formation of ubiquitin chains), where each additional ubiquitin is attached to the preceding lysine residue. The structure of these ubiquitin chains can vary significantly depending on the specific lysine involved in the linkage. The lysines used for chain formation include lysine 6 (K6), K27, K29, K33, K48, K63, and a unique Met1-linked (M1) ubiquitin (16, 17). The targeting of proteins for degradation by the proteasome is mainly achieved through the formation of K48 linkages. Conversely, K63 linkages can perform more diverse functions by changing the structure, location, or activity of the target protein (18, 19). M1 chains often serve as interaction platforms that facilitate signal transduction, including pathways such as NF-κB signaling (20) (**Figure 2B**).

Gastric cancer (GC) is a prevalent malignancy, ranking as the fifth leading cause of cancer-related mortality worldwide (21). The pathogenesis of GC involves a complex interplay of genetic and epigenetic alterations, similar to that of other malignancies. Key contributing factors include *Helicobacter pylori* infection and high salt intake, which play a significant role in the disease onset (22). Although patients with early-stage GC exhibit a favorable prognosis, those presenting with lymph node metastasis in advanced stages are predicted to have significantly reduced overall survival rates (23). The pathogenesis of human GC is notably intricate, necessitating a comprehensive understanding of its molecular mechanisms to develop effective treatment strategies aimed at improving patient survival rates. Investigating signaling pathways and identifying potential molecular targets associated with GC pathogenesis have emerged as critical approaches in the development of targeted therapies for the advanced stage of the disease (22). Several studies have identified key targets associated with GC, including DUBs.

In this review, the expression patterns of various DUBs in GC are discussed, along with their impact on patient survival rates. Additionally, this study emphasizes the regulatory function of USPs in the progression of GC.

## 2 Roles and mechanisms of DUBs in regulating GC

### 2.1 GC-promoting DUBs

#### 2.1.1 DUBs involved in regulating cell proliferation in GC

Proliferation, metastasis, and apoptosis are the three primary characteristics of cancer (24, 25). Dysregulation of the cell cycle can lead to alterations in cell proliferation, and E3 ligases are involved in nearly every stage of the cell cycle (26). Therefore, these ligases are expected to be regulated by DUBs.

**Figure 3:**
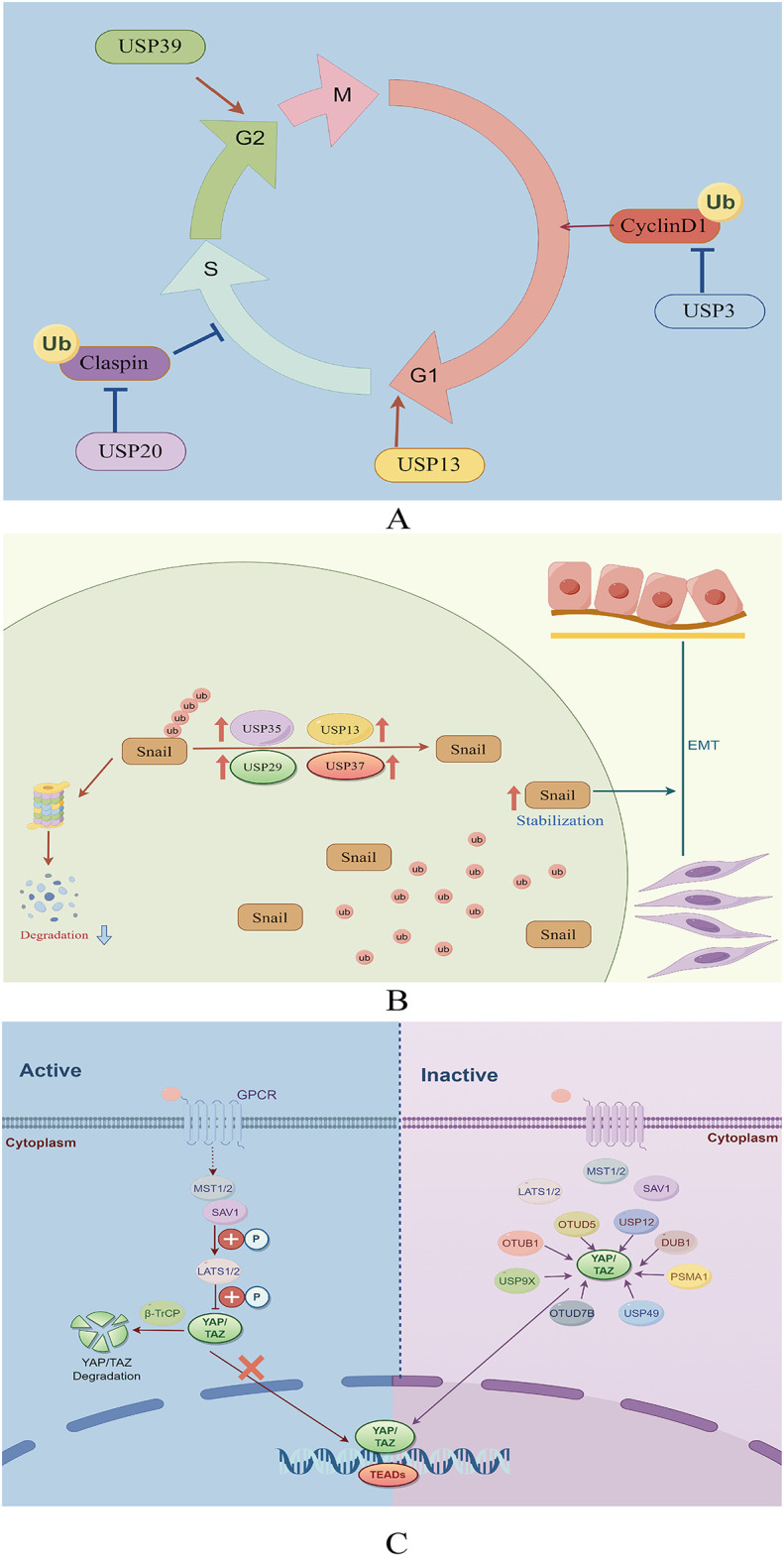
**(A)** Role of deubiquitinating enzymes on the cell cycle in GC. **(B)** The function of deubiquitinating enzymes in facilitating the EMT in GC. **(C)** The role of DUBs in the Hippo signaling pathway in GC.

USP13 removes ubiquitin molecules from cyclin D1, thereby stabilizing it and promoting cell cycle progression and proliferation in GC cells. Mechanistically, USP13 binds physically to the N-terminal domain of cyclin D1 and selectively deubiquitinates its K48-linked polyubiquitination chain while leaving K63-linked chains intact (27). Additionally, USP3 enhances the growth of GC cells by facilitating phase transition (from G1 to S) (28). Conversely, the suppression of USP39 expression reportedly impedes the proliferation of MGC-803 GC cells, leading to G2/M arrest (29). Furthermore, USP20 plays a role in regulating claspin stability, impacting the activation of cell cycle checkpoints (30). Reduced USP20 expression has also been associated with enhanced cell proliferation and accelerated progression from the G1 phase to the S phase of the cell cycle (30) (**Figure 3A**).

#### 2.1.2 DUBs in GC invasion and metastasis

Epithelial-mesenchymal transition (EMT) is essential in promoting the spread of cancer cells from the primary tumor to other locations (31). This complex process is coordinated by various EMT-promoting transcription factors, including Snail, Twist, and ZEB1 (32). USP13 promotes EMT by stabilizing Snail (33). Among the DUBs, USP29 is the most significant in preventing Snail degradation. The molecular mechanism underlying this stabilization involves the specific interaction between USP29 and SCP1, leading to the formation of a USP29/SCP1 complex that facilitates Snail deubiquitination (34). Snail1, a member of the Snail protein family, is characterized by its high instability and susceptibility to rapid degradation through the ubiquitin-mediated proteasome pathway during EMT (35). USP35 interacts with Snail1 to enhance its stability by removing its polyubiquitination chain. Additionally, USP35 promotes the invasion and migration of GC cells through its deubiquitinating activity (36). Pleomorphic adenoma gene like-2 (PLAGL2), a zinc finger plag transcription factor, activates the transcription of USP37, a DUB, which subsequently interacts with Snail1 to facilitate its deubiquitination. Targeting the PLAGL2-USP37-Snail1 pathway may represent a potential therapeutic strategy for GC through the inhibition of cell proliferation and migration (37) (**Figure 3B**).

#### 2.1.3 Hippo signaling pathway

The Hippo signaling pathway plays a crucial role in regulating cancer through the control of cell growth and proliferation (38). Activation of YAP/TAZ, the principal transcriptional activators of this pathway, occurs when cells detect favorable growth conditions, leading to their translocation into the nucleus (39, 40). Within the nucleus, YAP/TAZ interact with transcription factors to upregulate the expression of target genes, facilitating cell proliferation and suppressing apoptosis (39, 40). Therefore, dysregulation of the Hippo signaling pathway can promote tumorigenesis, underscoring the significance of regulating this pathway in GC treatment (41). YAP, as the principal downstream effector of the Hippo pathway, facilitates the expression of genes associated with cell proliferation and anti-apoptotic mechanisms through its interactions with transcription factors (42).

Recent research has underscored the significance of the Hippo-YAP1 pathway in the malignant progression of GC (43). However, investigations into the potential utility of YAP-specific inhibitors in cancer treatment remain limited. Analysis of data from The Cancer Genome Atlas (TCGA) revealed that YAP mutations and amplifications occur in only 4% of patients with GC, whereas over 50% exhibit high nuclear YAP expression (44). These findings suggest the significance of post-translational modifications in YAP activation, with ubiquitination serving as the primary post-transcriptional mechanism regulating YAP expression (45, 46). Moreover, certain DUBs have been reported to abnormally activate YAP by inhibiting its degradation via the UPS, thereby promoting GC progression.

USP49, a recently discovered DUB of YAP1, reportedly inhibits the proliferation, metastasis, chemoresistance, and peritoneal metastasis of GC cells when knocked down. Moreover, the YAP1/TEAD4 complex transcriptionally activates USP49, creating a feedback loop with YAP1 that promotes the malignant progression of GC cells (47). USP36, commonly referred to as DUB1, interacts with TAZ protein, facilitating its deubiquitination at various sites, notably at K48, thereby enhancing TAZ stability (48). In a recent study, OTUD5, a DUB of the OTU family, was demonstrated to mediate the proteasomal degradation of YAP1 (49). USP12 functions as an oncogene in GC, with its increased expression correlating with poor patient survival outcomes. Moreover, USP12 interacts with the YAP protein to inhibit its degradation in cancer cells, stabilizing YAP by impeding YAP K48-linked polyubiquitination at the K315 site of YAP (50).

Proteasome subunit alpha type 1 (PSMA1) is a key component of a multicatalytic proteinase complex characterized by a highly ordered, ring-shaped 20S core structure (51). Overexpression of PSMA1 in GC has been associated with a dismal prognosis. Subsequent studies have demonstrated that PSMA1 directly interacts with TAZ, inhibiting K27- and K48-linked TAZ ubiquitination. This interaction ultimately stabilizes and activates TAZ (52). YAP deubiquitination and stabilization by OTUB1 are essential for promoting cancer cell stemness and progression (53). In addition, a study revealed that OTUB1 binds to the YAP protein via its OTU region and removes ubiquitin from YAP at various lysine locations (K90, K280, K343, K494, and K497), thereby preventing YAP degradation (53). USP9X reportedly enhances cancer cell survival and reduces cell responsiveness to chemotherapy by deubiquitinating and stabilizing YAP (54). Zhang et al. also demonstrated that LINC01433, a long non-coding RNA associated with GC progression, enhances YAP stability by facilitating its binding with USP9X and also decreases YAP phosphorylation by weakening its bond with LAST1 (55) (**Figure 3C**).

#### 2.1.4 Other DUBs with Upregulated Expression in GC

##### 2.1.4.1 DUB

Increased USP1 levels have been observed in GC cell lines and patient samples, correlating with reduced survival rates. Suppression of USP1 expression reportedly impedes GC cell proliferation, migration, and invasion. Furthermore, USP1 facilitates metastasis by enhancing the stability of ID2 (56). Similarly, elevated USP11 levels have been associated with enhanced proliferation and migration of cancer cells, along with a decreased sensitivity to chemotherapy drugs. Mechanistic studies have also demonstrated that USP11 promotes GC progression by regulating the signaling pathways mediated by RhoA and Ras (57). High USP14 levels in stomach cancer have emerged as a potential prognostic marker for patient survival without disease recurrence (58). Furthermore, USP14 has been implicated in enhancing the growth, invasion, and migration of GC cells through the stabilization of the vimentin protein, which is crucial for EMT (59). Evidence suggests that USP15 knockdown hinders cell growth, invasion, EMT, and colonization in xenograft models. It affects glycolytic regulators through ubiquitination, reducing glycolytic activity and mitochondrial function. USP15 also regulates glucose metabolism by preventing the ubiquitination degradation of HKDC1 (60).

USP2 has been linked to cancer development through its facilitation of E2F4-mediated protective autophagy and maintenance of zinc levels. Therefore, inhibiting the USP2-E2F4 pathway could present a promising treatment strategy through the disruption of their interaction (61). Similarly, USP21 reportedly increases MAPK1 levels through the zinc finger transcription factor GATA binding protein 3, thereby promoting tumor progression and stem cell characteristics in GC (62). In addition, USP22 plays a crucial role in maintaining the pluripotency of GC cells by supporting BMI1 function. It also enhances cell growth and metastasis by activating the FOXO1 and YAP signaling pathways (63, 64). Lim et al. proposed that USP22 increases SOS1 levels in GC, thereby stimulating the Ras/Erk and Ras/PI3K/Akt signaling pathways (65). Furthermore, Deng et al. demonstrated that USP22 influences the Wnt/β-catenin signaling pathway by interacting with RNF220, facilitating the development of GC cells (66). Additionally, USP24 acts as a DUB for polo-like kinase 1 (PLK1), resulting in the activation of Notch-1 and promoting GC glycolysis and growth. Therefore, targeting the USP24/PLK1/Notch-1 pathway may present a promising therapeutic approach for GC (67). Zhao et al. discovered that USP28 is overexpressed in GC, thereby promoting tumor growth and metastasis by enhancing lysine-specific demethylase 1 levels (68). Moreover, USP29 expression was notably elevated in GC and correlated with a lower survival rate. *In vitro* and *in vivo* experiments have also demonstrated that USP29 enhances the progression of GC (69). Furthermore, FUBP1 regulates the transcription of the USP29 gene and interacts with AURKB to maintain its expression by inhibiting K48-linked polyubiquitination, thereby forming the FUBP1-USP29-AURKB regulatory pathway (69).

A study demonstrated that USP3 expression is upregulated in GC and enhances both proliferation and EMT *in vitro* and *in vivo*, correlating with poor patient prognosis (28). However, USP3 knockdown inhibits transforming growth factor-β-induced EMT. USP3 stabilizes the transcription factor Zeste 12 Homolog (SUZ12) through deubiquitination. Notably, SUZ12 knockdown inhibits USP3-induced GC cell migration and invasion, as well as EMT. Therefore, this USP3-SUZ12 axis may contribute to tumor progression, thereby presenting a potential target for therapeutic intervention in human GC (70). Additional research has demonstrated that USP3 increases the collagen protein levels of COL9A3 and COL6A5 by removing ubiquitin, thereby promoting EMT, as well as the invasion and migration of GC cells (71). Additionally, USP32 increases the levels of Smad2, a crucial protein in the transforming growth factor-β signaling pathway, stimulating the growth, metastasis, and chemoresistance of GC cells. However, the regulation of Smad2 expression by USP32 requires further investigation (72). As an upstream regulator of fatty acid synthase, USP38 deubiquitinates and stabilizes the expression of the fatty acid synthase protein, promoting the proliferation, migration, and tumorigenesis of GC cells (73). The deubiquitinating enzyme USP39 facilitates the proliferation and metastasis of GC cells by regulating the degradation of the RNA-binding protein RBM39 (74).

USP4 enhances the Warburg effect and promotes the growth of GC cells by deubiquitinating the M2 isoform of pyruvate kinase (75), regulating levels of phosphatases of regenerating liver-3, and activating the nuclear factor-kappa B (NF-κB) signaling pathway, thereby facilitating GC proliferation and metastasis (76). Hou et al. demonstrated a correlation between elevated USP42 levels and unfavorable outcomes in patients with GC (77). *In vitro* and *in vivo* studies have also demonstrated that the reduction of USP42 activity suppresses the metastasis of GC cells, indicating that targeting USP42 could be a viable therapeutic strategy (77). USP43 has been implicated in enhancing GC cell proliferation through the deubiquitination of stress-inducible protein 1 (78). Furthermore, USP44 expression in cancerous tissues is reportedly higher than that in normal gastric mucosa, suggesting that USP44 overexpression may be associated with DNA aneuploidy in malignancies. Notably, elevated USP44 expression serves as a negative prognostic marker for cancers characterized by DNA aneuploidy (79).

USP51 maintains ZEB1 levels, which activates ACTA2 transcription, triggering the mesenchymal characteristics of GC cells and enhancing tumor metastasis (80). USP54 reportedly interacts with PLK4 to remove ubiquitin, thereby increasing PLK4 protein levels. CEP120 promotes USP54 expression, underscoring the significance of the CEP120/UP54/PLK4 pathway in GC development (81). Elevated USP9X levels have been associated with unfavorable outcomes in patients with GC, indicating its potential as a cancer-promoting factor (82). Consistent with this data, another study demonstrated that USP9X suppression impedes the migration and invasion abilities of GC cells (83). Additionally, USP9X stabilizes MTH1, influencing GC cell proliferation, survival, migration, and invasion (84).

##### 2.1.4.2 OTU

Weng et al. demonstrated a correlation between elevated OTUB1 levels and more severe clinical characteristics in patients with GC, such as tumor invasion depth, lymph node involvement, and nerve invasion, which contributed to a decreased disease-specific survival rate (85). The interaction of GPX4 with OTUB1 prevents its ubiquitination and degradation, thereby enhancing its stability. This stabilization shields GC cells from ferroptosis and consequently promotes the progression and metastasis of GC (86). Similarly, the expression of OTUB2 has been demonstrated to be upregulated in GC tissues and cell lines, correlating with a negative prognosis (87). Notably, OTUB2 inhibition resulted in decreased growth, metastasis, and sphere formation in GC cells. Therefore, OTUB2 may function as an oncogene in GC by removing ubiquitin and enhancing the stability of epithelial keratin KRT80 through Lys-48-linked and Lys-63-linked deubiquitination (87). UCHL3 has been demonstrated to enhance the migration and invasion of GC cells by increasing IGF2 expression (88). A recent study revealed that UCHL3 attenuated the ubiquitination-mediated degradation of MTA2, thereby facilitating the progression of GC (89). Moreover, Epstein-Barr virus (EBV) is linked to various cancers, including EBV-associated GC. The EBV-encoded gene BALF1 has been shown to undergo degradation via the UPS, with OTUD1 identified as a key regulator of its stability (90). OTUD1 also enhances GC aggressiveness (90).

##### 2.1.4.3 JAMM

COP9 signalosome (CSN)5, also referred to as COPS5 or Jab1, functions as the catalytic component of the COP9 signalosome (91). CSN5 inhibition suppresses the growth of GC cells and triggers apoptosis through the regulation of p53 and Bax expression (92). Additionally, CSN5 triggers proteasomal degradation of the tumor suppressor p14ARF through a non-ubiquitin pathway (93). Similarly, CSN5 facilitates the nuclear export and subsequent degradation of another tumor suppressor, RUNX3 (94). In patients with GC, PTBP1 expression is significantly elevated and has emerged as a novel prognostic indicator. PSMD14, functioning as a DUB, enhances the growth, migration, and infiltration of GC cells by supporting PTBP1 stability (95). Moreover, BRCC3 expression is upregulated, triggering the PI3K/Akt/mTOR pathway, which results in malignancy (96).

##### 2.1.4.4 MJD

Studies have identified Ataxin-3 as a factor associated with GC development. Decreased ataxin-3 levels were observed in GC tissues and cells, correlating with clinicopathological features, such as tumor size, Lauren classification, histological differentiation, and p53 mutation status (97). However, the underlying molecular mechanisms remain largely unclear (**Table 1**).

**Table 1:** Deubiquitinases (DUBs) upregulated in gastric cancer (GC).

DUBs	Substrates in GC	Brief biological mechanisms	References
USP1	ID2	USP1 deubiquitinates, increases ID2 protein stability, and promotes GC progression.	(56)
USP11	/	USP11 enhances the effectiveness of chemotherapy in gastric cancer by RhoA and Ras signaling pathways.	(57)
USP14	Vimentin	USP14 removes ubiquitin from vimentin to promote cancer in gastric cells.	(59)
USP15	HKDC1	USP15 regulated glucose metabolism activity by inhibiting the ubiquitination degradation of HKDC1.	(60)
USP2	E2F4	The USP2-E2F4 axis can inhibit the autophagic machinery needed for zinc balance in gastric cancer growth.	(61)
USP21	MAPK1	USP21 deubiquitinates MAPK1 by interacting with GATA3, impacting gastric cancer growth and stem cell properties.	(62)
USP22	/	1.USP22 enhances gastric cancer advancement by influencing FOXO1 and YAP signaling pathways through c-Myc/NAMPT/SIRT1. 2.USP22 boosts SOS1 levels in gastric cancer, activating Ras/Erk and Ras/PI3K/Akt pathways. 3. USP22 connects with RNF220 to influence the Wnt/β-catenin pathway, promoting gastric cancer cell development.	(63–66)
USP24	PLK1	USP24 promotes tumor growth in gastric carcinoma by stabilizing PLK1 to activate NOTCH1 and increase aerobic glycolysis.	(67)
USP28		USP28 promotes the growth and spread of stomach cancer.	(68)
USP29	AURKB	Activation of USP29 by FUBP1 promotes the stability of AURKB and its oncogenic functions in gastric cancer.	(69)
USP3	SUZ12 COL9A3/COL6A5	1. USP3 enhances gastric cancer by deubiquitinating SUZ12. 2. USP3 enhances gastric cancer growth and metastasis by stabilizing COL9A3/COL6A5 through deubiquitination.	(70, 71)
USP32	Smad2	USP32 enhances gastric cancer growth and drug resistance by increasing SMAD2 levels.	(72)
USP38	FASN	USP38 enhances gastric cancer progression through deubiquitination and increases the stability of fatty acid synthase.	(73)
USP39	RBM39	USP39 promotes the growth and metastasis of gastric cancer cells by modulating the degradation of RBM39	(74)
USP4	PKM2	1. USP4 boosts gastric cancer cell growth and glucose metabolism by deubiquitinating and increases the stability of PKM2. 2. The USP4 enzyme acts as an oncoprotein in gastric cancer and controls NF-kappaB signaling by regulating PRL-3 expression.	(75) (76)
USP42	/	Promotes GC progression	(77)
USP43	STIP1	USP43 promotes gastric cancer progression by stabilizing STIP1 through deubiquitination.	(78)
USP44	/	USP44 is associated with DNA aneuploidy and can predict outcomes in gastric cancer.	(79)
USP51		USP51/ZEB1/ACTA2 promotes GC EMT.	(80)
USP54	PLK4	CEP120/USP54/PLK4 enhances centrosome amplification and gastric cancer advancement.	(81)
USP9X	MTH1	Hsa_circ_0008434/miR-6838-5p/USP9X promotes gastric cancer progression.	(82, 83)
		USP9X stabilizes MTH1 to promotes gastric cancer cell growth, survival, migration, and invasion.	(84)
OTUB1	GPX4	CST1 promotes gastric cancer metastasis by regulating GPX4 protein stability via OTUB1.	(85, 86)
OTUB2	KRT80	OTUB2 regulates KRT80 stability via deubiquitination and promotes gastric cancer growth.	(87)
UCHL3	MTA2	UCHL3 promotes gastric cancer metastasis by increasing IGF2 expression.	(88)
		UCHL3 reduced MTA2 ubiquitination degradation to promote GC progression.	(89)
OTUD1	BALF1	OTUD1 boosts gastric cancer aggressiveness by deubiquitinating the EBV protein BALF1.	(90)
CSN5	p14ARF	1.CSN5 contributes to gastric cancer growth by degrading p14ARF without using ubiquitin proteasomes. 2. CSN5 is involved in facilitating the degradation of the tumor suppressor RUNX3	(93, 94)
BRCC3	/	LncRNA TMPO-AS1/miR-126-5p/BRCC3 axis promotes gastric cancer growth and angiogenesis by activating the PI3K/Akt/mTOR pathway.	(96)
Ataxin-3	/	Promotes GC progression	(97)

### 2.2 Other DUBs with downregulated expression in GC

Cylindromatosis (CYLD) is a distinct K63 linkage-specific DUB within the USP family, notable for its lack of the zinc finger domain typically involved in distal ubiquitin interaction. Initially identified as a tumor suppressor gene linked to cylindromatosis, a condition characterized by multiple benign skin tumors, recent research has reported that CYLD negatively impacts various signaling pathways, including NF-κB, Akt, and Wnt (98–100). In the context of GC, CYLD expression is downregulated and correlates with poor clinical outcomes (101). Similarly, USP33 expression is reduced in GC tissues and cell lines, correlating with decreased patient survival rates. Low USP33 expression is also associated with greater tumor invasion depth and more advanced TNM staging. Cox regression analysis further identified USP33 as an independent prognostic marker for patient survival (102, 103). In addition, USP33 overexpression inhibits the proliferation, migration, and invasion of gastric adenocarcinoma cells (102). Xia et al. demonstrated that USP33 deubiquitinates and stabilizes Robo1, which is essential for the SLIT2-induced prevention of EMT and GC cell metastasis (103). Additionally, Yan et al. reported reduced BAP1 levels in GC, which was associated with more advanced tumor traits and lower survival rates, suggesting its function as a tumor suppressor (104).

### 2.3 Dual role of DUBs in GC

A20, also known as TNFAIP3, functions as a ubiquitin-editing enzyme, possessing both DUB and E3 ligase activities (105). A20 expression increases in GC tissues and cell lines (106). Notably, reducing A20 levels has been demonstrated to suppress the growth, spread, and infiltration of GC cells (106). Moreover, A20 has been identified as a target of miR-200a. Increased miR-200a levels or decreased expression of A20 promote apoptosis through decreased RIP1 polyubiquitination and enhanced caspase-8 cleavage (107). However, A20 has been reported to exhibit a two-fold function in GC, particularly in the context of *H. pylori* infection (108, 109).

A study revealed that UCHL1 could function as a tumor suppressor and a potential diagnostic biomarker for GC (110). However, contrasting findings from two additional studies reported elevated UCHL1 levels in liver metastases of GC, suggesting that increased UCHL1 expression may promote the growth, migration, and infiltration of GC cells (111). Mechanistically, activated Akt and Erk1/2 pathways are linked to the anticancer effect of UCHL1 (112). Given these conflicting results, additional research is required to investigate the precise role of UCHL1 in GC pathogenesis.

UCHL5 functions as a deubiquitinating enzyme associated with the proteasome; its elevated expression has been associated with improved survival in a specific subset of early-stage GC cases (113). However, contrasting evidence from another study suggests that UCHL5 enhances the stability of NFRKB, a chromatin-modifying protein, potentially facilitating the growth and metastasis of GC cells (114). These divergent findings underscore the need for further investigation into the precise biological role of UCHL5 in GC.

The function of USP15 in GC remains poorly characterized and contradictory. Zheng et al. suggested that USP15 might participate in suppressing the proliferation, migration, and infiltration of GC cells through deubiquitination of NF-κB, facilitated by the CSN complex, with a focus on stabilizing IκBα (115). Conversely, other studies have demonstrated that inhibiting USP15 suppresses Wnt/β-catenin signaling and impedes GC progression *in vitro* and *in vivo*; however, the precise mechanism by which USP15 regulates this pathway remains unknown (116). According to Das et al., USP15 can either stimulate or suppress the NF-κB and Wnt/β-catenin signaling pathways, depending on its interactions with different proteins in various cellular environments (117).

Zeng et al. demonstrated that USP10 expression was reduced in GC cell lines and clinical samples compared with those in non-cancerous cell lines and normal samples (118). The decreased levels of USP10 in patients with GC suggest the presence of severe clinicopathological characteristics and low survival rates, highlighting the potential of USP10 as a prognostic indicator (118). Furthermore, USP10 inhibits GC cell migration and invasion by suppressing EMT through TNFRSF10B deubiquitination (119). The calcium-binding protein S100A12 has also been identified as a prognostic indicator for GC, with its concentration correlating with USP10 levels (120). Despite the findings suggesting that USP10 functions as a tumor suppressor, other studies have indicated that USP10 may enhance the migration and invasion of GC cells through RFC2 stabilization (121). Consequently, the role of USP10 in GC remains unclear and requires further investigation (**Table 2**).

**Table 2:** Dual role of DUBs in GC.

DUBs	Roles	Brief biological mechanism	Reference
A20	Tumor suppressor	MiR-29a-3p promotes gastric epithelial cell migration by reducing A20 expression.	(87)
	Oncogene	Promotes GC progression	(106, 107)
UCHL1	Tumor suppressor	Inhibits GC progression	(110)
	Oncogene	UCHL1 promotes gastric cancer progression through Akt and Erk1/2 pathways.	(111, 112)
UCHL5	Tumor suppressor	UCHL5 expression in gastric cancer correlates with a better prognosis.	(113)
	Oncogene	lncRNA DRAIC/UCHL5/NFRKB axis promotes GC progression.	(114)
USP15	Tumor suppressor	USP15 suppressed GC cell proliferation, migration, and invasion.	(115)
	Oncogene	USP15 promotes gastric cancer progression through the Wnt/β-catenin pathway.	(116, 117)
USP10	Tumor suppressor	Inhibits GC progression by deubiquitinating TNFRSF10B.	(118, 119)
	Oncogene	CircCOL1A2/miR-1286/USP10/RFC2 axis promotes GC progression.	(121)

### 2.4 Role of DUBs in immune escape

One of the defining characteristics of cancer is its ability to evade the immune system (122). A key immune checkpoint involved in this process is CD274, also known as programmed cell death protein-1(PD-L1) or B7-H1, which regulates immune responses by binding to the PD-1 receptor on T cells, thereby inhibiting their activation (123). Increasing evidence suggests that deubiquitination plays a vital role in regulating PD-L1 protein levels and tumor cell immunosuppression (124). Therefore, targeting the ubiquitination/deubiquitination of PD-L1 could be a viable approach to enhance antitumor immunity. Studies have demonstrated that USP7 depletion leads to a reduction in PD-L1 expression in GC cells in a dose- and time-dependent manner (125). Additionally, USP7 elimination promotes the polyubiquitination and subsequent degradation of PD-L1, thereby increasing the susceptibility of cancer cells to T-cell-mediated cytotoxicity (125). These findings indicate that USP7 functions as an upstream DUB of PD-L1 in GC, counteracting its inhibitory effects on tumor growth by diminishing PD-L1-mediated immunosuppression. Furthermore, research indicates that ATXN3 may act as a positive regulator of PD-L1 transcript levels in GC (126), whereas CSN5 plays a role in stabilizing PD-L1 in GC cells, facilitating immune evasion (127). Similarly, studies have demonstrated that USP51 promotes ACTA2 transcription via ZEB1, leading to the activation and recruitment of fibroblasts and stimulating the polarization of M2-like macrophages (80).

### 2.5 Role of DUBs in cancer drug resistance

Drug resistance poses a significant concern in various diseases, particularly cancer. Emerging evidence suggests that drug resistance in cancer may be attributed to mechanisms such as drug efflux, epigenetic modifications, alterations in target molecules, changes in the metabolome, and mutations (128). Additionally, numerous studies have demonstrated that DUBs participate in chemoresistance. Cisplatin, a common chemotherapeutic agent, plays a crucial role in the treatment of various solid tumors and cancers, including GC, by primarily interacting with DNA, triggering apoptosis, impeding cell viability, and eliminating tumor cells (129). However, a significant challenge persists, as many initially responsive tumors develop resistance to cisplatin during treatment. USP32 expression is upregulated in GC tissues, correlating with diminished overall survival rates and advanced T stages in patients. Notably, USP32 suppression has been demonstrated to mitigate cisplatin resistance in GC cells. Furthermore, a study suggested that lncRNA CRAL functions as a competitive endogenous RNA that reverses cisplatin resistance in GC by modulating the miR-505/CYLD/Akt signaling pathway (130). Similarly, miR-20a has been observed to directly target CYLD and activate the downstream targets Livin and Survivin, potentially contributing to the development of resistance to cisplatin chemotherapy in GC (131). The exosomes released by tumor-associated macrophages contain miR-588, which interacts directly with the 3’ untranslated region of CYLD, leading to the suppression of its expression and loss of tumor suppressive function. This interaction also promotes increased proliferation and resistance to cisplatin in cancer cells (132). Moreover, the deubiquitinating enzyme USP7 stabilizes hnRNPA1, facilitating the secretion of exosomes derived from cancer-associated fibroblasts that transfer miR-522, thereby enhancing the resistance of GC cells to cisplatin chemotherapy (133). Furthermore, the expression of PSMD7, a component of the 19S regulatory subunit that operates independently of ATP hydrolysis and belongs to the JAMM family of metallopeptidases, is upregulated in GC cells. This upregulation results in increased cell proliferation, migration, and invasion, as well as enhanced resistance to cisplatin (DDP) through RAD23B ubiquitination and stabilization (134).

Oxaliplatin, a common chemotherapeutic agent for GC treatment, demonstrates efficacy in achieving remission; however, the development of resistance to oxaliplatin-based therapy persists among patients (135). The precise mechanisms underlying this resistance are not fully elucidated. *In vitro* investigations have revealed that miR-454 enhances GC cell proliferation and promotes resistance to oxaliplatin by directly targeting CYLD (136). Furthermore, the inhibition of USP10 mediates the deubiquitination of YBX1, resulting in a decrease in the expression levels of YBX1, promoting pan apoptosis in GC cells and reducing oxaliplatin resistance (137). Additionally, USP15 knockdown has been demonstrated to significantly impede cell proliferation and invasion, EMT while enhancing the antitumor effect of oxaliplatin (60) (**Table 3**).

**Table 3:** DUBs in cancer drug resistance.

Drugs	Brief biological mechanism	Reference
Cisplatin	Inhibiting USP32 can help overcome cisplatin resistance in gastric cancer cells.	(129)
	LncRNA CRAL/miR-505/CYLD/Akt can reverse cisplatin resistance in gastric cancer.	(130)
	miR-20a targets CYLD, potentially causing chemotherapy resistance in GC to cisplatin.	(131)
	TAM-released exosomes containing miR-588 suppress CYLD expression, promoting proliferation and resistance to cisplatin.	(132)
	USP7 stabilize hnRNPA1 through transferring miR-522 to increase gastric cancer cells’ resistance to cisplatin treatment.	(133)
	PSMD7 leads to GC cell growth, movement, and invasion, resistance to cisplatin, by promoting RAD23B deubiquitination and stability.	(134)
Oxaliplatin	miR-454 enhances gastric cell growth and reduces sensitivity to oxaliplatin by targeting CYLD.	(136)
	USP10 deubiquitinates of YBX1 leading to increased apoptosis in gastric cancer cells and decreased resistance to oxaliplatin.	(137)
5-FU	IU1 was able to reverse resistance to 5-FU in gastric cancer cells by inhibiting USP14 activity.	(58)
Camptothecin and etoposide	USP47 regulates gastric cancer cell viability and chemoresistance by activating the NF-κB signaling pathway.	(162)

### 2.6 Relationship between DUBs and *H. pylori*

*Helicobacter pylori* exhibits a predilection for colonizing the gastric mucosa and is implicated as a risk factor for chronic gastritis, inducing the development of precancerous lesions (138). In the chronic infection by *H. pylori* that progresses to malignancy, akin to persistent viral infections, certain bacterial effectors may play a crucial role in modulating host cell UPS pathways. The upregulation of USP35 expression in GC tissues may be partially attributed to *H. pylori* infection (36). Furthermore, a previous study demonstrated that *H. pylori* infection led to decreased expression and activity of USP7 in infected GC cells (139). However, the precise role of *H. pylori* in modulating USP7 expression remains uncertain. During *H. pylori* infection, the collaborative actions of USP48 and A20 were found to enhance the survival of *H. pylori*-infected GC cells, indicating a potential oncogenic role for A20 (109). Conversely, another study demonstrated that *H. pylori* infection led to miR-29a-3p upregulation, which facilitated the migration of gastric epithelial cells by downregulating A20 expression. This suggests that A20 silencing may induce EMT and promote the progression of GC (108). Therefore, further investigation into the role of A20 in *H. pylori*-related GC is required.

### 2.7 DUBs regulated by non-coding RNAs

Non-coding RNAs, such as circular RNAs, long non-coding RNAs (lncRNAs), and microRNAs (miRNAs), are significant in the regulation of GC (140). For example, cir_0017639 is reportedly upregulated in GC cell lines, leading to increased proliferation and migration by upregulating USP3 expression through miR-224-5p sequestration (141). Furthermore, Jin et al. demonstrated that the exosomal lncRNA SND1-IT1, secreted by GC cells, recruits the RNA helicase DDX54 to enhance the stability of USP3 mRNA and interacts with miR-1245b-5p to further upregulate USP3 expression (142). Cai et al. reported that the circHECTD1/miR-1256/USP5 axis activates the downstream β-catenin/c-Myc signaling pathway, facilitating tumor growth (143). CircRPS19 upregulates USP7, facilitating HK2 deubiquitination and subsequently restoring glucose consumption and lactate production (144). Cir_0008434, a miRNA sponge targeting miR-6838-5p, upregulated USP9X expression and facilitated the advancement of GC (83). circCOL1A2 sequestered miR-1286, leading to the downregulation of RFC2 ubiquitination levels through the upregulation of USP10 expression, consequently enhancing the invasive and migratory capabilities of GC cells (121). LINC00240 promotes malignant proliferation, migration, metastasis of cancer cells *in vivo* and *in vitro*, and the progression of GC by eliminating the ubiquitination of cancer protein DDX21 through its downstream DUB USP10 (145).

**Figure 4:**
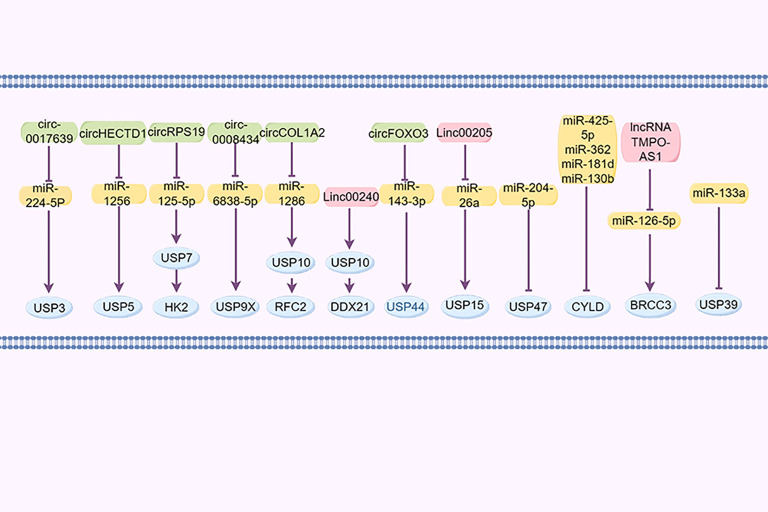
DUBs regulated by non-coding RNAs.

Huangfu et al. (146) posited that USP15 may contribute to the development of GC through its regulation by the LINC00205/miR-26a axis. Additionally, miR-133a reportedly suppresses USP39 expression by directly targeting its 3’UTR. circFOXO3 has been found to enhance the progression of GC by sequestering miR-143-3p, leading to an increased USP44 expression (147). Furthermore, miR-204-5p has been identified as targeting USP47 to inhibit its expression (148). miR-425-5p (149), miR-362, miR-181d, and miR-130b (150) downregulate CYLD expression in GC by binding to its 3’UTR region, promoting the progression of GC. BRCC3, as a member of JAMMs, was increased in GC and controlled by the lncRNA TMPO-AS1/miR-126-5p axis. TMPO-AS1 sequesters miR-126-5p to enhance the expression of BRCC3, consequently activating the PI3K/Akt/mTOR pathway and promoting malignancy (96) (**Figure 4**).

## 3 Bioinformatic analysis

### 3.1 Aberrant expression of DUBs and SUMO in GC

**Figure 5:**
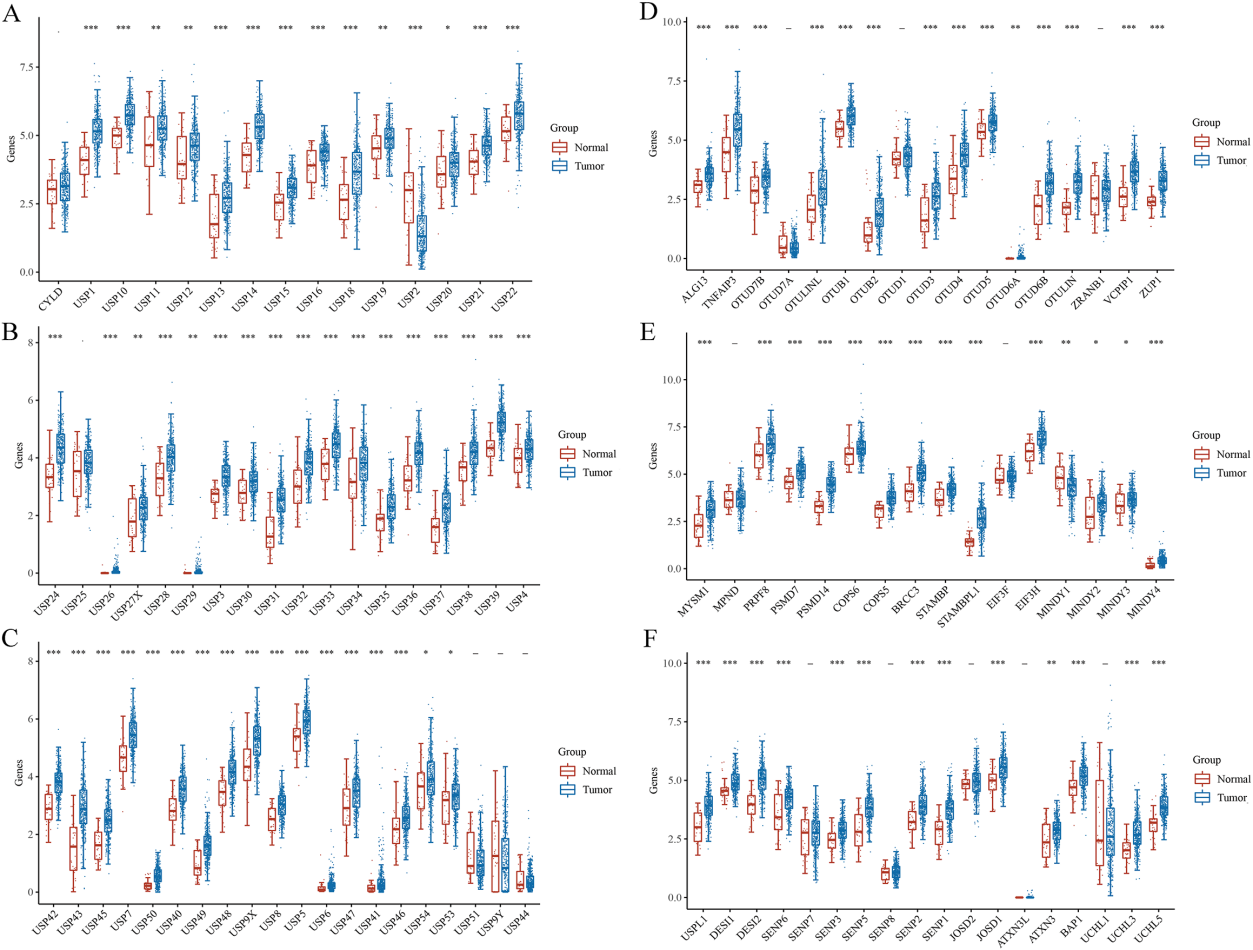
Gene and protein expressions of DUBs and SUMO in GC based on TCGA database. *P < 0.05, **P < 0.01, ***P < 0.001.

RNA sequencing (RNAseq) data, along with corresponding clinical information for GC, were obtained from TCGA database. In addition, DUB- and sumosylase-related proteins were analyzed using 375 GC and 32 normal control samples obtained from TCGA database (**Figure 5**). All raw data were pre-processed using the limma package in R software (4.1.3, R Foundation for Statistical Computing, Vienna, Austria). The expression profiles of DUB family members in GC and adjacent tissues are depicted in **Figures 5A–C**. Notably, members of the USP1s (USP1, USP11, USP12, USP13, USP14, USP15, USP16, USP17, USP18, and USP19) and USP3s (USP3, USP31, USP32, USP33, USP34, USP35, USP36, USP37, USP38, and USP39) were highly expressed in GC. Additionally, USP21, USP22, USP24, USP26, USP27x, and USP29 were highly expressed in GC. Similarly, USP4, USP41, USP42, USP43, USP45, USP46, USP47, USP48, USP49, USP5, USP6, USP7, USP8, and USP9x were highly expressed in GC.

Several members of the OTU family, including ALG13, TNFAIP3, OTUD7B, otulinl, OTUB1, OTUB2, OTUD3, OTUD4, OTUD5, OTUD6A, OTUD6B, OTULIN, VCPIP1, ZRANB1, were highly expressed in GC (**Figure 5D**). ZUP1 in the ZUFSP family was highly expressed in GC. Moreover, MYSM1, PRPF8, PSMD7, PSMD14, COPS5, COPS6, BRCC3, STAMBP, STAMBPL1, EIF3H, MINDY2, MINDY3, and MINDY4 in the JAMM family were highly expressed in GC (**Figure 5E**). BAP1, UCHL3, and UCHL5 in the UCH family were also highly expressed in GC. Similarly, USPL1, DESI1, DESI2, SENP1, SENP2, SENP3, SENP5, and SENP6 in the sumo family were highly expressed in GC (**Figure 5F**). Finally, JOSD1, JOSD2, and ATXN3 in the MJD family were highly expressed in GC.

### 3.2 DUBs and SUMO in regulating tumor immune responses

**Figure 6:**
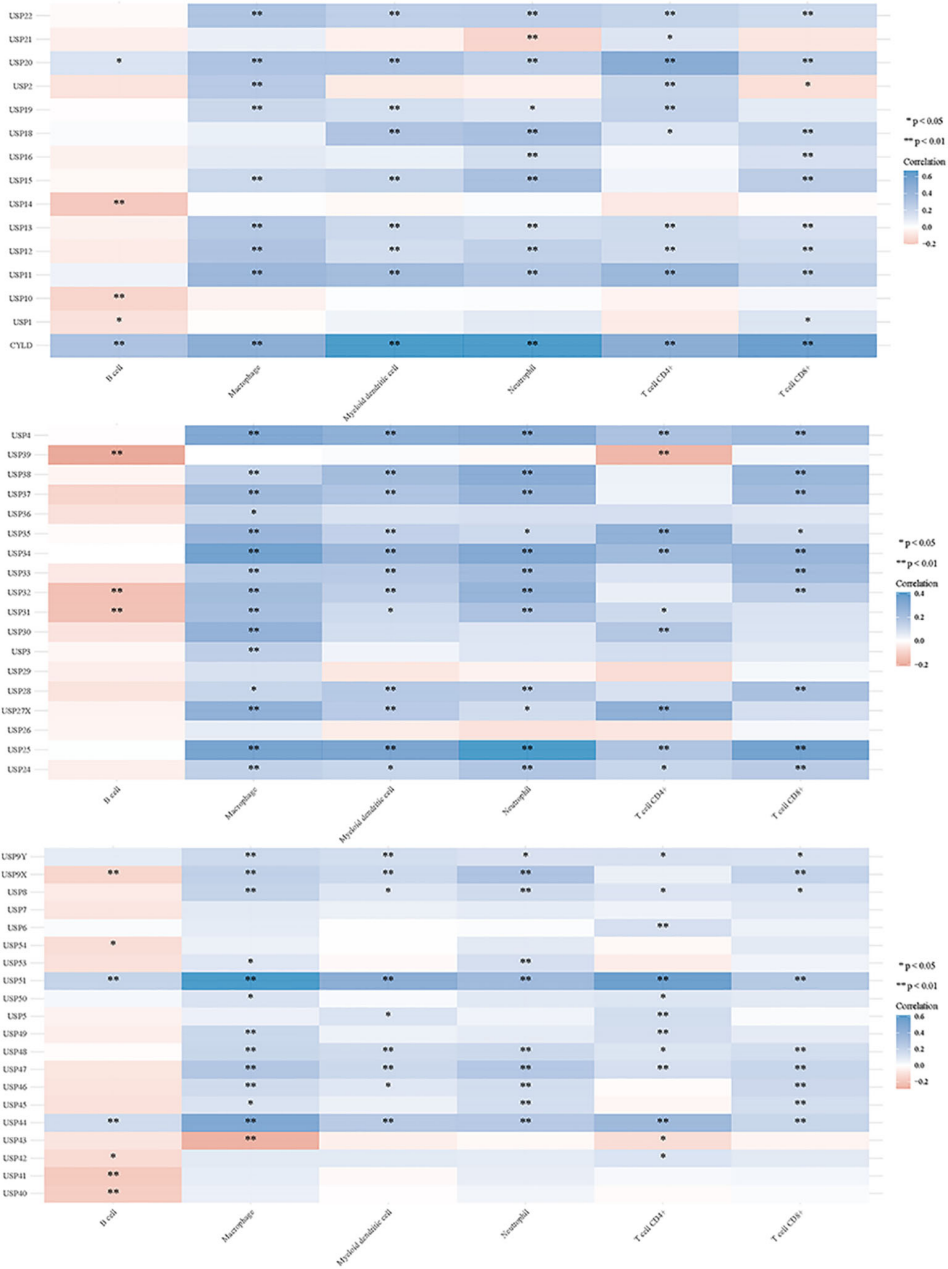
DUBs and small ubiquitin-like modifier proteins (SUMO) regulate the immune response of tumors.

**Figure 7:**
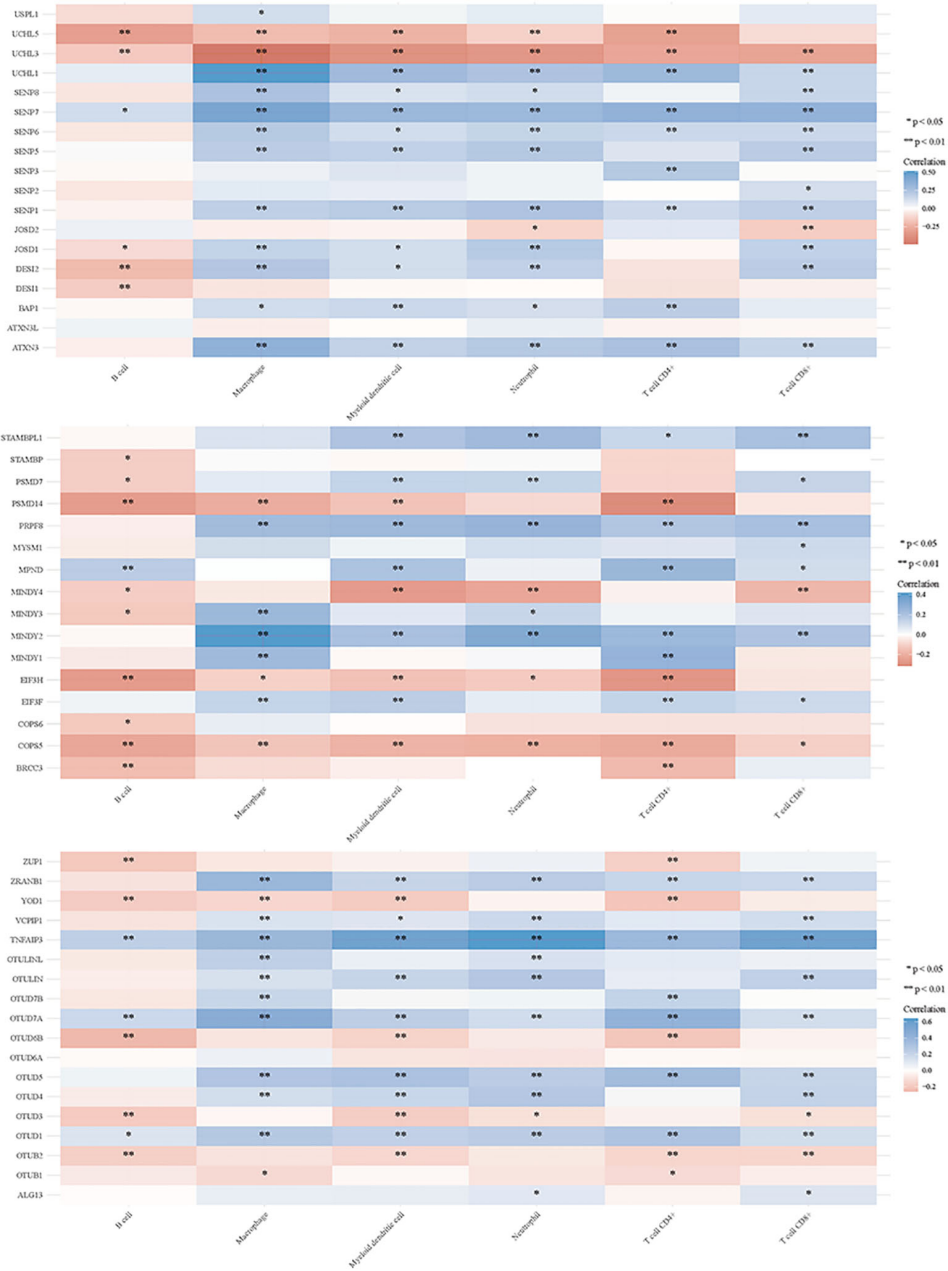
DUBs and SUMO regulate the immune response of tumors.

Deubiquitination modulates various signal transduction pathways, including some immune regulatory pathways (151). For this study, RNAseq data and corresponding clinical information for GC were obtained from TCGA. Additionally, data from TIMER2.0 (http://timer.cistrome.org/) were analyzed. Multi-gene correlations were visualized using the pheatmap package in R software. To further investigate the immune functions of DUBs and SUMO in cancer regulation, we assessed their correlations with the six studied immune cells (B cells, CD4+ T cells, CD8+ T cells, macrophages, neutrophils, and myeloid dendritic cells) (**Figures 6**, **7**). Notably, USP20, USP51, CYLD, TNFAIP3, OTUD7A, and OTUD1 were positively correlated with these immune cell populations; however, UCHL3 and COPS5 were negatively correlated.

## 4 Targeting therapy for GC

DUBs have been extensively investigated in various cancer types, including GC. Several studies have demonstrated that targeting this pathway may offer a potential therapeutic strategy for tumor treatment (152). For instance, Gavory et al. demonstrated that the inhibitor Almac4 selectively inhibits USP7, thereby enhancing the sensitivity of GC cells to T-cell-mediated toxicity, reducing proliferation, and inducing cell cycle arrest (125). Similarly, C9, a quinoline-4 (3H)-one derivative that inhibits USP7, suppresses GC cell growth by increasing the levels of p53 and its subsequent target p21 (153). Moreover, Compound 19, a derivative of (1–3) triazolo and [4, 5-d] pyrimidine, effectively inhibits the function of USP28, mitigating the deleterious effects on GC cells (154). In addition to degrading lysine-specific demethylase 1, Compound 19 targets c-Myc, another USP28 substrate (154). Furthermore, a different study demonstrated that the inhibitor IU1, which targets USP14, reduces the migratory and invasive capabilities of GC cells, inhibits cell growth, and promotes cell death (58).

## 5 Discussion

DUBs have been increasingly associated with tumor formation, particularly in the context of GC. This review outlines the mechanisms by which DUBs regulate the development and progression of GC. Most DUBs play a pro-cancer role in GC, promoting the proliferation and metastasis of GC cells while increasing their resistance to chemotherapy. Although many DUBs facilitate GC progression, some exhibit inhibitory effects, and others demonstrate dual roles. Notably, USP7, ATXN3, CSN5, and USP51 participate in tumor immunity within the GC microenvironment. DUBs are also involved in regulating non-coding RNAs during the biological processes of GC, highlighting the significance of the ncRNA-miRNA-DUBs axis in GC progression.

The regulation of DUBs in GC is complex, involving multiple pathways and targets. Targeted therapies are crucial for treating patients with high DUB expression. Currently, molecularly targeted drugs and small molecule inhibitors for ubiquitination and deubiquitination enzymes are used in cancer treatment (9, 155). This study reviews the inhibitors associated with GC-related DUBs, highlighting the extensive research surrounding USP family inhibitors. Although small molecule inhibitors for USP14, USP7, and USP28 have been identified, their development remains in the nascent stages. Developing specific USP inhibitors poses significant challenges, with a risk of off-target effects owing to the involvement of USPs in various biological processes. Recently proteolysis-targeting chimeras (PROTACs) have emerged as a potential alternative cancer therapeutic strategy (156, 157). PROTACs may enhance drug sensitivity and minimize side effects compared to small-molecule inhibitors (158). Various PROTACs have been developed using different E3 ligases. Each PROTAC consists of a ligand that binds to the target protein at one end and another that connects to the E3 ligase, facilitating the ubiquitination and degradation of the target protein by the proteasome (159). Unlike traditional small molecule inhibitors that occupy the active site, PROTACs employ transient binding through the UPS to ubiquitinate and eliminate target proteins (160, 161).

Research on Machado–Joseph domain-containing proteases (MINDY) and the role of DUBs in GC is limited, presenting significant opportunities for further exploration. Future studies on DUBs may enhance the understanding of GC pathogenesis and treatment. Furthermore, the development of targeted drugs and small molecule inhibitors is expected to become a central focus of research, potentially offering new avenues for GC treatment.
